# Remarks on Belladonna

**Published:** 1849

**Authors:** E. S. Cooper

**Affiliations:** Peoria Ill.


### ARTICLE IV.

*Remarks on Belladonna as an application to Ulcers.* By E. S. Cooper M. D. of Peoria Ill.

Although no one remedy can be said to be applicable to all ulcers or even the same ulcer, if indolent at differant periods, in a much as articles which prove most beneficial lose their influence after several applications, and have to be superceded by others, the extract of Belladonna has proven eqully adapted to many cases, and all stages of the same case, does not appear to lose its stimulus after repeated application and in that has proved superior to any article I have used. If granulations are exuberant it gives tone to their vessels, and very soon changes their color, and morbid growth, if languid it stimulates them to increased growth, and has proven applicable to more cases and of greater benefit than any article I have used in all ulcers

In many cases of ulcers about the nail in which as Hunter says, the parts are not in a state of harmony, it is with great difficulty as is well known, they are made to heal. Remove the nail which was black, apply the black lotion and lint, with the internal use of Sarsaparilla decoction and in a few days the parts begin to granulate—Granulations florid, and to all appearance every thing is going on well, cicatrization even commences and bids fair to continue to the completion of a cure when, however, suddenly a nail, black as the first, changes entirely the condition of the sores, and in a few days it looks as badly as at any time previously. Remove this nail and it goes through the same course as before, which will continue for weeks and even months, in spite of every effort, local and constitutional, to bring the sore into a healthy state. Nor are hese the worst of this kind of ulcers, for in many cases they never show a disposition to heal from the time the nail has been removed until it reappears, and during the time be extremely offensive. Practitioners who have met with many of these cases, will not be suprised at my dwelling so long upon a matter apparently so unimportant. It is in these cases particularly I would recomend the extract of Belladonna. The following are very similar to many others in which I have used the article.

Case I. Master S. æt. 7., bruised the end of his thumb in May, 1842. In June I was consulted, and found the dorsal side of the thumb ulcerated for three fourths of an inch from the end, including absorption of most of the nail. The excavation much deeper in the space previously occupied by the nail. The nail was divided into two portions, which were short, curled backwards, and jet black. The sore had been in that condition three weeks.

I removed the nail, and applied calomel with lime water, and lint, and as the patient was debilitated I gave Columba and Sarsaparilla in decoctiou.

*July 1st.* Ten days since I saw the patient—Ulcer the same as before, patient's health somewhat improved, sore very offensive, bleeds when touched a little roughly, considerably painful—Added one fourth grain of Ox. Mur. Mercury to a pint of decoction and continued it, applied Ox. Mur. Mercury to Ulcer, also lint, and covered with oil silk.

4th. Surface of Ulcer pale and secreting considerably, very offensive, continued decoction as before. Applied Ox. Mur. Mercury with bals. fir, lint, and oil silk.

8th. Still very offensive and pale as before. Discontinued Ox. Mur. Hyd., both externally and internally, continue decoction. Applied Nitric Acid Solution with lint and oil silk.

12th. Ulcer about the same, continued the treatment.

16th. Considerably painful last night, discharges largely, and is very offensive. Ordered blue pill and opium at night. Applied Carb. Ferri and Acet. Plumb. and, instead of oil silk and lint, used linen merely.

This was continued for near a week when a new nail appeared, or rather two portions of nail, one on either side, which were attached at the root. This also curled as the first, and was black. I removed it and afterwards used various articles trying first lotions, then ointments, afterwards simple lint, and continued for four weeks when a third nail appeared. This I dissected out, with the gland which produced it, from which I hoped to derive some benefit, but to my utter chagrin six weeks passed, and although no appearance of nail to prevent a cure, the sore seemed obstinately the same, and during the time I had tried many articles which are recommended and some not recommended, and at last applied extract of belladonna, more for experiment as I had tried many others, with out any particular hopes of its proving beneficial.

The patient had suffered much pain in the arm at times before the use of this artcle which was occasionally so severe as to prevent sleep, but after its application for two or three days he suffered no pain, and in two weeks florid granulations had arisen a little above the surrounding surface, and in four weeks cicatrization was completed. The use of the article was not intermitted from the commencement, until the cure was effected. The extract was moistened a little and spread into the form of a covering, which was reapplied twice per week, and was all the dressing the ulcer received. It appeared alike effiecient in allaying the pain of the part, and reducing the sore to a healthy condition.

Case II. I was consutled in January 1845, in reference to an ulcer on the index finger of Miss M. æt. 11, who had it injured six months previously by a strike from a hammer. It had been sore two months when a physician was called who applied a blister which removed the nail, gave the patient some blue pill, and applied mercurial ointment, which soon brought the sore into a healthy state, small florid granulations covered the surface and a speedy cure was anticipated which was, however finally interrupted, by the growth of the nail, which was black. thin, and curling. This was taken away with forceps having apparently but slight attachment, and the wound afterwards treated with the application of Sub. Mur. Hyd. and Aqua Calc., which appeared to have a good effect, and the sore looked to be in a good condition as before, which continued until the appearance of the nail again. It then assumed its former unhealthy aspect, the granulations become absorbed, and the excavation became deeper than before including part of the bone. But the nail was permitted to remain some weeks before its removal this time, which, how ever being finally effected the sore became healthy again, and remained so until the reappearance of the nail. After which it became as ill-conditioned as before. And it was at this time I was consulted.

Remembering the former case, after removing the nail, I made the application of extract belladonna as before and with like result. The gland not being dissected out the nail was reproduced but instead of the black color, it was of the redish hue which marks newly reproduced cuticle, and which afterwards remained a shade redder than the balance of the nails.

Several other cases have occured in my practice, similar to these and although the good effects of the belladonna were not so striking they were sufficient to convince me that it was superior to most or all other articles with which I was acquanted in ill-conditioned ulcers. One advantage it certainly does posess over other applications to sores viz., it never loses its influence, the sore never becomes accustomed to its action, and I have witnessed its good effects for weeks in allaying the pain of malignant mammary ulcers, after the disease was so far advanced as to exclude all hope of cure. I have never known an individual complain of severe pain in an ulcer to which it was applied.

